# Remote ischemic preconditioning to reduce contrast-induced acute kidney injury in chronic kidney disease: a randomized controlled trial

**DOI:** 10.1186/s12882-018-1169-x

**Published:** 2018-12-22

**Authors:** Ali Ghaemian, Jamshid Yazdani, Soheil Azizi, Ali A. Farsavian, Maryam Nabati, Alireza Malekrah, Mozhdeh Dabirian, Fatemeh Espahbodi, Bahareh Mirjani, Hossein Mohsenipouya, Javad Heshmatian

**Affiliations:** 1 Department of Cardiology, Cardiovascular Research Center, Mazandaran Heart Center, Artesh BLVD, Sari, Iran; 2 Faculty of Health Sciences, Mazandaran University of Medical Sciences, Sari, Iran; 3 Department of Pathology, Mazandaran University of Medical Sciences, Sari, Iran; 4 Department of Internal Medicine and Nephrology, Mazandaran University of Medical Sciences, Sari, Iran; 5 Cardiovascular Research Center, Mazandaran Heart Center, Artesh BLVD, Sari, Iran

**Keywords:** Remote ischemic preconditioning, Acute kidney injury, Contrast-induced nephropathy, Coronary angiography

## Abstract

**Background:**

The impact of contrast-induced acute kidney injury (CI-AKI) on patients with chronic renal disease is well-known. Remote ischemic preconditioning (RIPC) is a non-invasive method that can reduce the risk of CI-AKI, but studies on RIPC have had different results. The aim of the present study was to assess the potential impact of RIPC on CI-AKI.

**Methods:**

In a randomized, double blinded, controlled trial, 132 patients with chronic renal dysfunction (glomerular filtration rate < 60 mL/min/m^2^) who underwent coronary angiography or angioplasty received adequate hydration. RIPC was performed in 66 patients by applying an upper arm blood pressure cuff. The cuff was inflated four times for 5 min to 50 mmHg above the systolic blood pressure, followed by deflation for 5 min. In the control group, the blood pressure cuff was inflated only to 10 mmHg below the patient’s diastolic blood pressure. The primary endpoint was an increase in serum cystatin C ≥ 10% from baseline to 48–72 h after exposure to the contrast.

**Results:**

The primary endpoint was achieved in 48 (36.4%) patients (24 in each group). RIPC did not show any significant effect on the occurrence of the primary endpoint (*P* = 1). In addition, when the results were analyzed based on the Mehran risk score for subgroups of patients, RIPC did not reduce the occurrence of the primary endpoint (*P* = 0.97).

**Conclusions:**

In patients at moderate-to-high risk of developing CI-AKI when an adequate hydration protocol is performed, RIPC does not have an additive effect to prevent the occurrence of CI-AKI.

**Trial registration:**

The clinical trial was registered on (Identification number IRCT2016050222935N2, on December 19, 2016 as a retrospective IRCT).

## Background

Diagnostic coronary angiography (CA) and percutaneous coronary intervention (PCI) are routinely performed in patients with coronary artery disease (CAD). Contrast-induced acute kidney injury (CI-AKI) increases mortality and morbidity, and it is one of the most important adverse effects of contrast medium (CM), particularly in patients with pre-existing renal dysfunction [1, 2]. Based on the Kidney Disease Improving Global Outcome guidelines, AKI is defined as a 30% rise or 0.3 mg/dl increase in serum creatinine level [3] The European Society of Urogenital Radiology defined contrast-induced nephropathy (CIN) as an increase of the serum creatinine level ≥ 0.5 mg/dL or > 25% of the baseline value 48–72 h after CM administration [4]. Cystatin C has been suggested to be more sensitive than serum creatinine to detect acute changes in renal function [5, 6]. In addition, Briguori et al. demonstrated that a serum cystatin C increase ≥10% from that at baseline was the best marker to identify patients at risk of developing CIN [7]. Remote ischemic preconditioning (RIPC) has been shown to have protective effects on the remote organs by alternating ischemia and reperfusion and releasing mediators in the body [8, 9]. Recently, RIPC has been evaluated to prevent CI-AKI [10–12]. Although RIPC showed protective effects on urinary liver-type fatty acid-binding protein levels in patients with CI-AKI at low and moderate risks of developing CIN, the serum creatinine levels in the RIPC and control groups did not show significant differences [10]. On the other hand, a pilot study reported by Er et al. showed a reduced incidence of CIN in high-risk patients, based on serum creatinine levels measured 48–72 h after CM administration [12].

Given the lack of enough data regarding the protective effects of RIPC in patients at moderate-to-high risk of developing CIN and the different effects of RIPC on serum creatinine and cystatin C levels in various reports, we performed a double-blind, randomized, controlled trial to assess the impact of RIPC after CM administration to protect renal function.

## Methods

We performed a prospective, randomized, single-center, double-blinded, sham-controlled trial between December 2016 and August 2017 at Mazandaran Heart Center, Mazandaran University of Medical Sciences, Sari, Iran. All patients gave written informed consent and the study is registered at http://www.irct.ir/ IRCT2016050222935N2.

### Subjects

Patients with stable CAD who were admitted to the hospital for PCI were selected if the estimated glomerular filtration rate (eGFR) was< 60 ml/min/1.73 m^2^, according to the Modification of Diet in Renal Disease formula [13, 14]. Among patients who were admitted to the hospital for CA, those with an eGFR< 60 ml/min/1.73 m^2^ were selected if their estimated CM consumption was> 100 mL. Patients in whom the contrast volume was < 100 mL or > 5 × body weight (kg)/serum creatinine (mg/dL) mL were excluded [15].

Patients on hemodialysis or peritoneal dialysis, those < 20 years old, those with any abnormality or problem in their arms, and those who did not provide informed consent were also excluded. Finally, 140 patients out of 222 patients with an eGFR< 60 mL/min/1.73 m^2^ were included in our study. For random allocation of patients into control or RIPC group, we used random number table and simple randomization was performed. For randomization, we used sealed envelopes for patients to receive either sham preconditioning (control) or RIPC in a 1:1 ratio.

### Study protocol

Nephrotoxic drugs such as metformin, non-steroidal anti-inflammatory drugs, and aminoglycosides were discontinued. In our department, to treat patients with renal dysfunction, we routinely use N-acetylcysteine 600 mg twice orally and hydrate patients with an intravenous infusion of saline 0.9% solution at a dose of 1 ml/kg/h for 12 h before through 12 h after CA or PCI. We performed this protocol for all study patients.

In the study group, we applied a standard blood pressure cuff to the patients’ upper arms. RIPC was performed with four cycles of 5-min inflation, followed by 5-min deflation of the cuff at 50 mmHg above the patient’s systolic blood pressure.

In the control group, the four cycles of 5-min inflation and deflation of the blood pressure cuff were performed, but only at10 mmHg below the patient’s diastolic pressure. The diagnostic or therapeutic procedure was performed within 40 min of performing RIPC or the sham intervention. Both the patients and the interventional cardiologist were blinded to the inflation pressure interventions that were performed before the procedure.

The primary endpoint was the increase of serum cystatin C ≥ 10% from baseline to 48–72 h after CM administration. The secondary endpoints were the incidence of CI-AKI (defined as a rise of the serum creatinine level ≥ 0.3 mg/dL) and the level and change in serum creatinine and cystatin C levels within 48–72 h after CM exposure. Hemodialysis, readmission, and mortality within 2 months of contrast administration were our clinical endpoints.

We used Ultravist 300 (Iopromide; 300 mg iodine/mL; Bayer Pharmaceutical, Germany) isoosmolar CM for all patients. Blood samples were taken at baseline before the procedure and 48–72 h after contrast administration.

### Statistical analysis

We calculated the sample size on the basis of the pilot study by Er et al. [12]. On that study the mean peak change from baseline of cystatin C in control group was 121.43% ± 22% vs 106.7% ± 6% in RIPC group. We considered the significant level and power of the study to be 5 and 90%, respectively. In addition, considering 20% sample loss, the calculated number of intervention and control group were 60 patients. In this study, all continuous variables were tested for normal distribution using the Kolmogorov-Smirnov test. Variables with normal distribution are presented as a mean ± standard deviation. For variables that were not normally distributed, we used the median and first and third quartiles (Q_1_-Q_3_). Normally distributed continuous variables were analyzed using Student’s t-test, and the Mann-Whitney *U* test was used to compare non-normally distributed data. Fisher’s exact and chi-square tests were used to compare qualitative variables such as the patients’ gender. We used SPSS 20 for the statistical analysis, and a probability value< 0.05 was considered statistically significant.

## Results

**Fig. 1:**
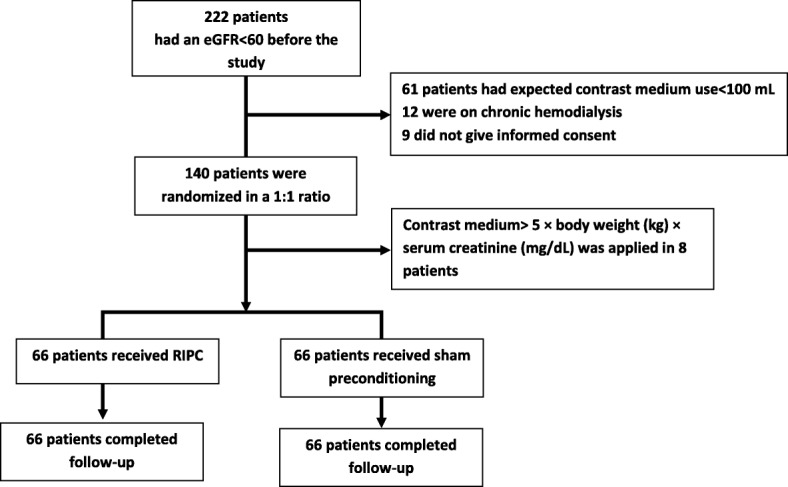
Study flow chart

During the study period, we assessed 222 patients with a baseline eGFR< 60 mL/min/1.73 m^2^for potential recruitment (Fig. 1). In 61 patients, the expected contrast medium use was< 100 mL, 12 were on chronic hemodialysis, and 9 did not agree to enroll in the study. A total of 140 patients were included for randomization to receive either RIPC or sham preconditioning in a 1:1 ratio. After randomization, 8 patients were excluded because their CM use was> 5 × body weight (kg)/serum creatinine (mg/dL) [7].Finally, 132 patients, (66 in the RIPC group and 66 in the control group) were included in the study, and all of them completed the study protocol and follow-up procedures. The demographic and other clinical baseline characteristics of the two groups did not show significant differences, except the RIPC group had a higher frequency of coronary artery bypass graft (CABG) surgery than the control group did; 10(15.2%) participants in the RIPC group vs 3(4.5%) in the control group (*P* = 0.04) (Table 1).

**Table 1:** Baseline characteristics

	Control group *n* = 66	RIPC group *n* = 66	*P*-value
Age (years)	65.27 ± 8.9	66.15 ± 8.63	0.57
Men, n (%)	42 (63.6)	51 (77.3)	0.13
Body mass index (kg/m2)	26.6 ± 3.5	26.6 ± 4.2	0.94
Underlying disease n (%)			
Diabetes	29 (43.9)	27 (40.9)	0.73
Insulin therapy	3 (4.5)	4 (9.1)	0.49
Hypertension	42 (63.6)	41 (62.1)	0.86
Smoking	19 (28.8)	19 (28.8)	1
Dyslipidemia	31 (47)	28 (40.9)	0.48
Peripheral artery disease	2 (3)	7 (10.6)	0.16
Prior coronary artery disease	32 (48.5)	38 (56.1)	0.38
Prior myocardial infarction	10 (15.2)	10 (15.2)	1
Prior percutaneous coronary intervention	4 (6.1)	4 (6.1)	1
Current percutaneous coronary intervention	15 (22.7)	12 (18.2)	0.52
Prior coronary artery bypass graft surgery	3 (4.5)	10 (15.2)	0.04
Laboratory data			
Baseline eGFR (mL/min/1.73 m^2^)	42.77 ± 9.46	41.86 ± 8.16	0.55
Baseline hemoglobin (g/dL)	12.44 ± 1.94	11.91 ± 1.98	0.12
Baseline cystatin C (mg/dL)	1.42 (1.1–1.86)	1.58 (1.3–2)	0.17
Baseline serum creatinine (mg/dL)	1.5 (1.4–1.6)	1.5 (1.4–1.73)	0.42
Volume of contrast medium (mL)	100 (80–116)	90 (80–112.5)	0.35
Left ventricular ejection fraction	50 (53.75–55)	50 (40–55)	0.88
> 45%	50 (75.8%)	46 (69.7%)	
30–44%	10 (15.2%)	9 (13.6%)	
< 30%	6 (9.1%)	11 (16.7%)	0.97
Integer CI-AKI risk score			
Mean (Q_1_-Q_3_)	9 (6–13)	10 (7–13.25)	0.3
≤ 5	9 (13.6%)	4 (6.1%)	
6–10	30 (45.5%)	30 (45.5%)	
≥ 11	27 (40.9%)	32 (48.5%)	0.3

*RIPC* remote ischemic preconditioning, *eGFR* estimate glomerular filtration rate, *CI-AKI* contract-induced acute kidney injury

The baseline laboratory data, including serum cystatin C, serum creatinine, eGFR, and serum hemoglobin levels, were also similar between the two groups. The baseline data showed that all 132 patients had an eGFR< 60 mL/min/1.73 m^2^, and 125 patients (61 in the control group and 64 in the RIPC group) had a serum creatinine level > 1.4 mg/dL (*P* = 0.44). The frequency of current and previous PCI, volume of CM use, and probability of developing CI-AKI according to the Mehran risk score [16] were similar between the two groups. Different components of the Mehran risk score were also similar between two groups (Table 2). Most of the patients in both groups were categorized as having a moderate or high risk of developing CI-AKI. We also used the National Cardiovascular Data Registry (NCDR) risk score model [17] for predicting acute kidney injury in our patients and interestingly the risk of acute kidney injury and dialysis were not statistically different between the two groups (Table 3). In addition, the cardiovascular medications were also similar between the two groups (Table 4).

**Table 2:** Components of the Mehran Risk Score

Risk Factors	Risk score		
	Control group	RIPC group	*P*-value
eGFR < 60 ml/min/1.73 m^2^	2.26	2.28	0.95
Serum Creatinin > 1.5 mg/dl	0.93	0.65	0.27
Diabetes	1.3	1.48	0.49
CHF	1.93	2.04	0.78
Anemia	1.83	2.03	0.4
Contrast media volume	1.36	1.42	0.59
Age > 75 years	0.52	0.68	0.53

*CHF* congestive heart failure, *eGFR* estimate glomerular filtration rate

**Table 3:** NCDR AKI and Dialysis Risk after PCI

	Control group *n* = 66	RIPC group *n* = 66	*P*-value
AKI score	24 (17–43)	30 (21.5–43)	0.35
Risk of AKI	6.7 (4.9–21.7)	16.5 (6.7–21.7)	0.35
AKI requiring dialysis score	3 (2–6)	4 (2–6)	0.69
Risk of dialysis	0.15 (0.09–0.84)	0.27 (0.09–0.84)	0.67

*NCDR* National Cardiovascular Data Registry, *AKI* acute kidney injury, *PCI* percutaneous coronary intervention, *RIPC* remote ischemic preconditioning

**Table 4:** Baseline Cardiovascular Medications

	Control group *n* = 66	RIPC group *n* = 66	*P*-value
ß-blocker n (%)	42 (62.12)	44 (66.67)	0.72
Calcium channel blocker n (%)	14 (21.21)	12 (18.19)	0.66
Angiotensin-converting enzyme inhibitor n (%)	16 (24.24)	11 (16.67)	0.73
Angiotensin II receptor blocker n (%)	35 (53.03)	33 (50)	0.28
Loop diuretics n (%)	35 (53.03)	38 (57.57)	0.6
Thiazide diuretics n (%)	11 (16.67)	9 (13.6)	0.63
Spironolacton n (%)	5 (7.57)	7 (10.6)	0.55

The primary endpoint of the study (increase in cystatic C level ≥ 10% 48–72 h after CM administration relative to its baseline value) was achieved in 48 (36.36%) out of 132 patients; interestingly, its frequency was exactly the same in the two groups (24 participants in each group) (Table 5). Deterministic sensivity analysis was performed including 8 patients whom were excluded based on contrast dose. Observed odds ratio was 1 and the extended adjusted odds ratio and bias percent were 1.019 and 1.9%, respectively. The Mehran risk score-based subgroup analysis showed that there was no significant difference between the groups in the increase in cystatin C level ≥ 10% (*P* = 0.97) (Table 6). CI-AKI occurred in 4 (6.1%) participants in the control group and 2 (3%) in the RIPC group, and this difference was not significant (*P* = 0.68). The Mehran score in 4 of these 6 patients was ≥11, and the scores of the other 2 patients were 5 and 6. There was no statistically significant difference between the two groups in serum cystatin C and serum creatinine levels and their changes 48–72 h after the procedure (Table 5).

**Table 5:** Trial outcomes

	Control group *n* = 66	RIPC group *n* = 66	*P*-value
Primary endpoint			
Increase in cystatin C ≥ 10% after 48–72 h, n (%)	24 (36.4)	24 (36.4)	1
Secondary endpoints			
Contrast-induced acute kidney injury, n (%)	4 (6.1)	2 (3)	0.68
Serum creatinine after 48–72 h (mg/dL)	1.4 (1.25–1.53)	1.4 (1.2–1.54)	0.92
Change in serum creatinine after 48–72 h (mg/dL)	−0.11 (−0.3 to 0)	−0.165 (−0.31 to 0)	0.53
Cystatin C after 48–72 h (mg/L)	1.4 (1.12–1.74)	1.55 (1.22–2)	0.13
Cystatin C change after 48–72 h	+ 0.065 (− 0.185 to + 0.3)	− 0.01 (− 0.357 to + 0.387)	0.59
Clinical endpoints			
Re-admission within 2 months n (%)	18 (27.3)	16 (24.2)	1
Dialysis within 2 months n (%)	0	0	n.a.
Mortality within 2 months n (%)	1 (1.5)	0	0.69

*RIPC* remote ischemic preconditioning

**Table 6:** Mehran risk score, based on an increase in cystatin C ≥ 10%

Mehran risk score	Control *n* = 24 (36.36%)	RIPC group *n* = 24 (36.36%)	*P*-value
< 5	2	0	
6–10	12	11	
≥11	10	13	0.97

*RIPC* remote ischemic preconditioning

Dialysis was not required for any patient, and only one patient in the control group died after CABG surgery due to wound infection during the follow-up period. Thirty-four patients (18 [27.3%] in the control group and 16 [24.2%] in the RIPC group) were readmitted to the hospital (*P* = 0.69). None of the patients had any adverse effects due to the preconditioning protocol.

## Discussion

In this study, RIPC, which induces a short duration of ischemia in the upper arm before exposure to CM in diagnostic and therapeutic coronary procedures, did not demonstrate a protective effect to reduce CI-AKI in patients with chronic renal impairment who were at moderate-to-high risk of developing CIN. In addition, RIPC did not reduce adverse clinical outcomes including mortality, hemodialysis, and re-hospitalization.

Excretion of creatinine in urine is the result of glomerular filtration and tubular secretion [18]. Thus, its changes may underestimate the eGFR. Furthermore, the levels of serum creatinine depend on non-renal factors such as muscle mass and hydration status [19]. On the other hand, serum cystatin C does not undergo tubular secretion, and its serum concentration is determined by glomerular filtration [20, 21] and therefore it can reliably represent impairment of renal function [6, 22]. Briguori et al. studied 410 patients with renal dysfunction (eGFR< 60 mL/min/1.73 m^2^) who underwent coronary or peripheral procedures and showed that a cystatin C increase ≥10% compared with baseline was the best cut-off value to identify patients at risk of developing CIN [7]. In the same study, while the rate of CI-AKI based on serum creatinine rise ≥0.3 mg/dL was 8.2%, a serum cystatin C increase of ≥10% occurred in 21.2% of the patients.

Our study’s results are similar to and different from those of previous randomized trials involving RIPC to prevent CIN. First of all, similar to the study by Menting et al. [14] (72 patients) and Igarashi et al. [13] (60 patients), our study showed that RIPC did not significantly affect the change in serum creatinine 48–72 h after CM exposure. Furthermore, the change in serum cystatin C in both our study and that in the study by Igarashi et al. were not significantly different between the RIPC and control groups. Most patients in these two previous studies were at mild-to-moderate risk of developing CIN. However, in contrast to our study, Menting et al. found that the creatinine change in patients at high to very high risk of developing CI-AKI was significantly lower in the RIPC group compared with those in the control group. However, only 11 (15.3%) of their patients (6 in the RIPC group and 5 in the control group) had a Mehran risk score ≥ 11, while in our study 59 (44.7%) patients (27 in the control and 32 in the RIPC group) had a risk score ≥ 11. Moreover, Menting et al. did not measure the cystatin C level in their study.

On the other hand, the investigation by Er et al. [12], which included 100 patients (50 in each group), found that in patients with chronic kidney disease and a high risk of developing CI-AKI, RIPC reduced the incidence of CIN, defined as an increase in serum creatinine ≥0.5 mg/dL within 48–72 h after CM exposure. Although 59% of the patients in the study by Er et al. were at a high or very high risk of developing CI-AKI, the incidence of CIN in the control group was as high as 40%, whereas in our study, only 4 (6%) participants in the control group and 2 (3%) in the RIPC group had an increase in serum creatinine ≥0.3 mg within 48–72 after CM exposure.

Unfortunately, Er et al. did not report the incidence of cystatin C increase in their study; however they reported the protective effect of RIPC on the rise in serum cystatin C level [12]. Furthermore, the incidence of CI-AKI reported by Er et al. is much higher than that from another investigation with a comparable baseline eGFR [23]. Nonetheless, the authors suggested that this discrepancy may be the result of a high prevalence of heart failure and diabetes in their study cohort [12]. Finally, in contrast to the study by Er et al., in our study, RIPC did not affect the rates of re-hospitalization, death, or hemodialysis. As far as we know, no other investigation has reported that RIPC has a protective effect on the clinical adverse effects of CI-AKI in humans.

This study has some strengths. First, it was performed in a tertiary referral heart hospital, and the patients were initially included in the study based on their renal dysfunction, so the protocol represents the routine daily practice in our hospital. This is why more study cohort patients had a moderate or high risk of developing CIN. Second, this randomized controlled trial included more patients than previous similar studies did. Third, all patients were adequately hydrated according to the routine of our department 12 h before to 12 h after CM application. Thus, even though the incidence of the increase in serum creatinine was not high, the increase in cystatin C ≥ 10% was a more sensitive marker than serum creatinine, and it was sufficiently high to be comparable between the two groups. Finally, this study was completely double-blinded in order to reduce the risk of bias.

Nonetheless, the study has some limitations. First, the incidence of the increase in serum creatinine was low. However, all patients were hydrated based on a long schedule protocol. The incidence of CIN with adequate hydration has varied between 2 and 13% [24–26]. Second, the follow-up period of this study was only 2 months. An increase in serum cystatin C ≥ 10% was reported to predict the occurrence of major adverse events, defined as death and further deterioration of renal function requiring chronic dialysis at one year [7]. However, the purpose of this study was only to define the effect of RIPC on AKI induced by CM. Third, we did not examine creatinine clearance and urinary neutrophil gelatinase-associated lipocalin levels. Both of these markers are helpful to detect CI-AKI.

Finally, in this study patients received three prophylactic measures in the study group vs two in the control arm. In this way, the effect of RIPC for CI-AKI may be diluted. However, hydration of such patients was performed based on current guidelines and we could not deprive them from such prophylactic measures.

## Conclusions

The results of this study show that in patients at moderate-to-high risk of developing CI-AKI who are adequately hydrated, RIPC does not have further beneficial effects. Since there is no signal of actual benefit of RIPC on preventing AKI it seems that it is time to abandon it as a prophylactic measure for preventing CI-AKI. However, a large study only in high-risk patients (eGFR < 30 ml/min) should be performed to assess the effect of RIPC as an adjunct to hydration to provide additional protection in this high risk group.

## Abbreviations

CA: Coronary angiography
CABG: Coronary artery bypass graft
CI-AKI: Contrast-induced acute kidney injury
CIN: Contrast-induced nephropathy
eGFR: Estimated glomerular filtration rate
PCI: Percutaneous coronary intervention
RIPC: Remote ischemic preconditioning
